# Adaptation measures to sustain indigenous practices and the use of indigenous knowledge systems to adapt to climate change in Mutoko rural district of Zimbabwe

**DOI:** 10.4102/jamba.v10i1.388

**Published:** 2018-04-12

**Authors:** Shingirai S. Mugambiwa

**Affiliations:** 1 Department of Sociology and Anthropology, University of Limpopo, South Africa

## Abstract

This article examines adaptation measures used to sustain indigenous practices and the use of indigenous knowledge systems (IKS) to adapt to climate change in Mutoko rural district of Zimbabwe. Community-based adaptation is able to reduce the vulnerability as well as improve the resilience of the local people to climatic variability and change. Subsistence farmers have always adopted adaptive strategies to some of these changes over the years. As such, the adoption of indigenous practices will significantly help rural community members to adapt to climate change. This study employed a qualitative method and an exploratory design, and the results are derived from 30 purposively selected in-depth interviews. The study discovered that there are numerous measures used to adapt to climate change and subsequently to sustain indigenous practices. The study also found that the community no longer grows maize in large quantities, having shifted to millet and sorghum in order to adapt to climate change. The community also provided various strategies to adapt to climate change. These strategies include mulching, creating large storage houses for produce and creating temporary walls on riverbanks in order to store water when the rivers dry up. This study concludes that climate change adaptation measures employed by the community have significantly helped them to sustain their indigenous practices in many ways. Also, the use of IKS, through activities such as crop type change from maize to traditional millet and sorghum (which facilitates traditional lifestyle and activities), re-establishes the community’s indigenous practices since they are made to observe the practices of yesteryear.

## Introduction

This article examines the adaptation measures used to sustain indigenous practices and the use of indigenous knowledge systems (IKS) to adapt to climate change in Mutoko rural district of Zimbabwe. Adapting to climate change strategies are responses to actual or expected climatic stimuli which are meant to reduce harm or exploit associated beneficial opportunities. The adjustments can be categorised as either responses to current occurrences in the climate or the planned adaptation to long-term climate changes (Hisali, Birungi & Buyinza 2011). Adapting to climate change embraces adjustments in behaviour that reduce the vulnerability of society to climate hazards (Brenkert & Malone 2005; Brooks & Adger 2005; Smith, Ragland & Pitts 1996; Yohe & Tol 2002). Gyampoh et al. (2014) observe that rural communities that are vulnerable to climate change have strong adaptive capacities. Adapting to drought, scarcity of rain and decreased production of crops is accomplished through community-based measures to sustain human livelihoods. People in rural communities have established culture-based mechanisms of adaptation to harsh weather conditions over the years. As a result, such mechanisms play a significant role as adaptation measures which are employed to sustain indigenous practices and to adapt to the effects of climate change.

## Background and motivation

An adaptation to the impacts of climate change is accomplished through community-based measures to sustain human livelihoods (Bhusal 2009; Rankoana 2016). The mechanisms developed by rural communities are complex, are used within cultures and depend on the use of indigenous knowledge in the production of subsistence crops. People’s knowledge of the seasons motivates them to grow subsistence crops with a careful consideration of the soil fertility and texture as well as crop variations which enhance the sustainable production of crops. Madzwamuse (2010) asserts that small-scale farmers produce crops through a knowledge of environmental conditions, without the use of modern scientific knowledge. These adaptation methods are a product of the communities’ priorities, knowledge and capacities which allow them to plan and cope in the midst of climate change. The present study examined rural community members’ knowledge of climate change and their ability to adapt to its impacts on the community. Given the use of community-based strategies used by rural communities, as presented by other writers, this study seeks to establish community-based measures to sustain human livelihoods and the use of IKS to adapt to climate change particular to the Mutoko community. Smit and Wandel (2006) assert that the widely known purpose of adaptation studies in the climate change field is to estimate the degree to which anticipated or actual impacts of climate change scenarios could be moderated or mitigated. Hence, understanding indigenous adaptation methods employed by the community will significantly help in the moderation and mitigation of climate change impacts.

Climate variability risks have always been part of agricultural activities, such that in most cases African farmers have survived and coped with its impacts (Mano & Nhemachena 2007; Ziervogel et al. 2008). Wahaa et al. (2013) found traditional sequential cropping system to be the strategy used by most farmers in sub-Saharan Africa. In the study, they discovered that farmers grow the sequential cropping system most frequently applied in their district, composed of two short-growing crop cultivars. These two are single-cropping systems where farmers only grow one long-growing cultivar of the first crop of the traditional sequential cropping system; and the highest-yielding sequential cropping system where farmers grow the sequential cropping system composed of two short-growing crop cultivars with the highest yields. Furthermore, in a study by Gwimbi (2009), more than 65% of cotton-producing farmers in Gokwe Rural District (GRD) of Zimbabwe reported that in order to cope with climate change, they depend on the use of irrigation, a diversification into more drought-resistant crop varieties, a diversification into other crops and the timing the planting period to coincide with the onset of the rains.

## The concept of climate change adaptation

Climate change adaptation has been described in different ways by various scholars. However, the varying conceptualisations allude to the same process. Smit and Wandel (2006) assert that the application of the term ‘adaptation’ can be traced to Julian Steward, an anthropologist who used ‘cultural adaptation’ to describe the adjustment of ‘culture cores’ to the natural environment through subsistence activities. Denevan (1983:401) in Smit and Wandel (2006) considers (cultural) adaptation as a ‘process of change in response to a change in the physical environment or a change in internal stimuli, such as demography, economics and organization’. Cultural practices are equated with genetic characteristics in the natural sciences; in this Darwinian view, a group that does not have adequate methods of adapting to environmental stress will face serious challenges when competing for scarce resources and will fail to continue. As a result, a cultural practice is an ‘adaptation’ only if it developed to overcome stress, thereby distinguishing adaptations from ‘adaptive features’ that allow societies to function within their environments regardless of whether or not they evolved as a result of selection. Furthermore, Ford (2012) indicates that empirical work conducted in the Arctic describes adaptations employed to cope with a rapidly changing environment with a strong focus on food systems and the dangers of engaging in harvesting activities. Adaptations are underpinned by traditional knowledge of lands and resources, cultural identity and strong social and kinship networks within health systems that combine allopathic and traditional approaches to tackle the risks of climate change. A number of studies also describe community-based initiatives to raise an awareness of climate change impacts and to use research to empower communities. There are examples in the Arctic of the development of surveillance infrastructure for climate-related health outcomes. Few studies, however, have examined how non-Arctic groups are responding to the health effects of climate change, and how economic resources, institutions, technology, equity, information and skills affect this ability.

Smit et al. (2000) indicate that adapting to climate change and variability has been subjected to more intensive inquiry. As a result, analysts have seen the need to distinguish types, to characterise attributes and to specify applications of adaptation. For example, adaptation can refer to natural or socio-economic systems and be targeted at different climatic variables or weather events. Burton (1992) in Smit et al. (2000) defines the adaptation to climate change as the process through which people reduce the adverse effects of climate change on their health and well-being and take advantage of the opportunities that their climatic environment provides. Also, Smith et al. (1996) in Smit et al. (2000) define the adaptation to climate change as all the adjustments in behaviour or economic structure that reduce the vulnerability of society to changes in the climate system. Bradshaw, Dolan and Smit (2004) are of the view that adaptations come in many forms and can be characterised according to a suite of attributes such as intent, timing, duration, spatial extent or responsibility.

## Climate change adaptation strategies

An adaptation to climate change strategies are actions taken by people in response to climatic stimuli which are meant to exploit associated beneficial opportunities. The adjustments can be categorised as either responses to current occurrences in climate or planned adaptation to long-term changes in climate (Hisali et al. 2011). Adaptation allows for adjustments in a system’s behaviour and characteristics that enhance its ability to cope with external stresses. Given constant levels of hazard over time, adaptation allows a system to reduce the risk associated with these hazards by reducing its social vulnerability (Brooks & Adger 2005). Several lessons can be learnt from sociology and anthropological perspectives on human adaptation to climate change. Scientific studies in the disciplines of Anthropology and Sociology explored the mechanisms of adaptation to changing living conditions as a result of climate change (Brenkert & Malone 2005; Yohe & Tol 2002). Gyampoh et al. (2014) observe that rural communities that are vulnerable to climate change have strong adaptive capacities. An adaptation to drought, scarcity of rain and decreased production of crops is accomplished through community-based measures to sustain human livelihoods (Gyampoh et al. 2014). In a study conducted in Pakistan by Abid et al. (2016), farmers reported that the switch-over from traditional cotton varieties to genetically modified cotton varieties was the result of heavy pest attacks on traditional cotton varieties. They also reported a higher use of heat-tolerant wheat varieties in response to an increase in the frequency of extreme maximum temperature events. Another traditional method implemented by these farmers was the use of crop types that differed from previously used varieties and which were effective against the incidence of substantial pest and insect attacks. Some farmers also replaced cotton crops with maize (Abid et al. 2016).

Community-based adaptation is helpful in reducing the vulnerability as well as improving the resilience of the local people to climatic variability and change. Even though subsistence farmers have always adopted adaptive strategies to some of these changes over the years, effective adaptation strategies should be aimed at securing their well-being in the face of climatic changes (Somah 2013). Nhemachena and Hassan (2007) argue that the climate is already changing, to such an extent that adaptation is critical and of concern in developing countries, particularly in Africa where vulnerability is high. Mitigation plans may be implemented, but they would not be sufficient enough to avoid changes in the global climate, hence the importance of adaptation (Akoh et al. 2011:24). Furthermore, Gbetibouo (2008:1) states that the ‘extent to which the adverse impacts of climate change are felt depend on the extent of adaptation; without adaptation, climate change would be detrimental’. The Assessments of Impact and Adaptations to Climate Change (AIACC) project that was undertaken in various regions of Africa, Asia, Central and South America, the Caribbean and the Indian and Pacific Oceans found that overall there was an adaptation deficit, which is likely to be widened because of climate change (Leary et al. 2007). ‘Adapting to climate change will entail adjustments and changes at every level, from community to national and international levels’ (UNFCCC 2007:29). Burton, Smith and Lenhart (1998) are of the view that adaptation can take different forms depending on the action taken in response to the external threat. This implies that if the action is taken before the threat, the adaptation is preventive; and if the action is taken during the time of the threat, the adaptation is gradual and short term. Correspondingly, if the action is taken after the threat, the adaptation is reactive or corrective.

The ability of individual households and communities to adapt to climate change depends on their adaptive capacity. ‘Adaptive capacity refers to the potential or ability of a system, region, or community to adapt to the effects or impacts of climate change’ (Eriksen, O’Brien & Rosentrater 2008:18). This capacity is dynamic and is influenced by economic and natural resources, social networks, institutions, governance, human resources and technology. In most cases, the poor and the marginalised have a low adaptive capacity. Climate variability risks have always been part of agricultural activities, such that in most cases African farmers have survived and coped with its impacts (CCAFS 2009; Mano & Nhemachena 2007; Ziervogel et al. 2008). Ziervogel et al. (2008) attest that:

agricultural adaptation taking place in Africa is responding more to perceived climate variability rather than climate change, such that these responses are likely to be overwhelmed by climate change and its longer-term implications. (p. 21)

Adaptation measures in agricultural practices include crop and livestock variation, community-based adaptation, water storage, irrigation, rainwater harvesting, water conserving techniques and the use of drought-resistant crop varieties. In Zimbabwe, rainfall projections point to a drying trend, hence adaptation strategies in the agricultural sector should focus on strategies to conserve moisture, particularly adopting improved short-season seed varieties and drought-resistant small grains (Ministry of Environment and Tourism 2006).

In Ghana, farmers have always adopted a range of adaptation strategies, such as permanent and seasonal migrations, new crop varieties and irrigation practices as a result of increasing incidence of climate-related shocks and stresses (Tambo 2016). There are various adaptation measures employed by households to enhance their resilience to the changes in climate in Ghana. The most common adaptation measure which is practiced by 95% of the people is changing the planting dates (Tambo 2016). Similarly, in a study by Wahaa et al. (2013) on adaptation strategies, most farmers from different countries in sub-Saharan Africa also use changing the planting dates as an adaptation measure. Tambo (2016) provided some of the most prominent measures that are used in Ghana. These include the use of drought-tolerant or early maturing crop varieties, mixed cropping, crop switching and tree planting which are normally practiced by livestock farmers who plant shade trees to protect their livestock during heat stress. Wahaa et al. (2013) found traditional sequential cropping system to be the strategy used by most farmers in sub-Saharan Africa. In the study, they discovered that farmers grow the sequential cropping system most frequently applied in their district, composed of two short-growing crop cultivars. These two are single-cropping systems where farmers only grow one long-growing cultivar of the first crop of the traditional sequential cropping system; and the highest-yielding sequential cropping system where farmers grow the sequential cropping system composed of two short-growing crop cultivars with the highest yields. In the same study by Wahaa et al. (2013), in 35% of the surveyed districts, one or more sequential cropping systems exist. The sequential cropping systems frequently applied are mostly based on groundnut and maize as well as cassava, rice and wheat. However, few sequential cropping systems exist with sunflowers or soybeans, which are of minor importance in the surveyed households.

The literature on climate change adaptation around the world as presented in this article demonstrates that in various regions of Africa, Asia, Central and South America, the Caribbean and the Indian and Pacific Oceans, there is an adaptation deficit. This calls for an investigation into adaptation strategies employed in other parts of the world in order to increase adaptive capacity through imitation, where applicable. Nevertheless, in most studies, for instance, in Pakistan and Ghana, farmers employ strategies such as crop change, permanent or seasonal migrations and new crop varieties and irrigation practices. Also, the literature reveals that adaptation to climate change is determined by adaptive capacity, and this capacity is influenced by economic and natural resources, government, human resources and technology. However, this study goes beyond adaptation, as presented in other studies around the world, to focus on adaptation measures employed to sustain indigenous practices and the use of IKS to adapt to climate change. Also, unlike other studies on adaptation to climate change, the current study looks beyond government, institutions and technology as sources of adaptive capacity. Rather, it looks at the community-based adaptation strategies that are crafted and employed by the community to adapt to the effects of climate change.

## Theoretical framework

### Climate change adaptation theories

#### Marxist theory, capitalism and climate change

A social theoretical reflection of climate change is centred on several essential classic problems. Szerszynski (2010) asserts that sociologists are interested in presenting how climate change is viewed as a topic of scientific concern. The relationship between nature and culture creates an enduring attraction. In sociology and other related disciplines, Marxist theory is increasingly being deployed to examine the phenomenon of climate change. In this examination, emphasis is placed on how capitalism and its attendant processes are intractably linked to climate change. The view that Marxist scholars advance is that capitalism might have a hand in driving climate change, especially in underdeveloped countries. Urry (2010) asserts that climate change forms new positions of tension and contradiction in contemporary capitalism. Consequently, statistics suggest that more than 2.8 billion people are vulnerable to climate change and variability in socio-economic terms. Developing and underdeveloped countries are the most affected by the phenomenon (Global Humanitarian Forum [GHF] 2009). This fact calls for the need to inquire into the knowledge of climate change as well as methods that communities in the developing and underdeveloped communities are using to cope with climate change. Therefore, what Marxist theory does is to present a new perspective on how climate change can be linked to global processes unfolding within society.

#### The ‘risky society’ thesis

Within the discipline of sociology, Ulrich Beck’s thesis on ‘risky society’ provides a foundational basis for examining the changes unfolding in contemporary society. The theory attributes the changes unfolding within society to the project of modernity. Thus, at the centre of Beck’s analysis is the way a new form of rationality, centred on the role of science and technology that governs modern society, is creating certain environmental risks to populations. Applied to the sociological study of climate change, Beck’s theory sheds light on how scientific and technological development (rationality) are taken as explanatory variables to account for the emergence and rise of climate change (Beck 1992). Rather than pointing to the darker side of modern scientific and technological advancement, Beck highlights that modern society has to address the risks that come with modern scientific and technological change related to terrorism and climate change, among other disasters. However, despite both the Marxist theory and the ‘risk society’ thesis being some of the major climate change adaptation theories, their emphasis on adaptation is largely on the use of modern scientific and technological progress. Therefore, because of this limitation, the current study identified Afrocentricity as the most appropriate theoretical perspective.

#### Afrocentricity

Afrocentricity emerged from the Afrocentric paradigm which deals with the aspects of African identity from the perspective of African people. This concept has been termed ‘Afrocentricity’ by Molefe Asante in an effort to convey the profound need for African people to be relocated historically, economically, socially, politically and philosophically (Mkabela 2005). The theory became a growing scholarly idea in the 1980s as a large number of African American and African scholars adopted an Afrocentric orientation to data. The paradigm is generally opposed to theories that alienate Africans in the periphery of human thought and experience. The Afrocentric paradigm was adopted to understand how Mutoko rural community members explain the use of indigenous practices to adapt to climate change. The Afrocentric paradigm provides methods and practices that African people can use for making sense of their everyday experiences from an indigenous African’s point of view. The aim is to be sufficiently detailed and sensitive enough to actual social contexts so as to investigate the methodological bases or orderly character of ordinary social activities (Mkabela 2005). Local communities have developed culture-based mechanisms of adaptation to harsh weather conditions that negatively impact their livelihoods (Jianchu et al. 2007). These mechanisms are complex and were developed and are used within cultures which are in line with Afrocentricity. They imply a greater dependability on the use of indigenous practices to sustain their lives (Elia, Mutala & Stilwell 2014).

Furthermore, Afrocentricity seeks to answer the question, ‘What would African people do if there were no white people?’ This implies the question, ‘What natural responses would occur in the relationships, attitudes towards the environment and kinship patterns, among other aspects of life, for African people had there not been any intervention of colonialism or enslavement?’ In this light, Afrocentricity places the fundamental role of the African subject within the context of African history and in so doing eliminates Europe from the heart of the African reality. Hence, Afrocentricity is evidently a revolutionary idea since it studies numerous aspects of life that comprise ideas, concepts, personalities and political and economic processes placing African people as subjects rather than objects. Moreover, as a paradigm, Afrocentricity emphasises the significance of the African; that is, African ideals, values, culture and history which are of paramount importance in African culture (Kershaw 1990; Keto 1995). Asante (1993) argues that Afrocentricity has a significant impact on the manner in which Africans view their identity and way of doing things, such as farming practices and other indigenous practices they employ in their day-to-day lives. Africans have been sidelined from the social, political, philosophical and economic aspects of life terms. Hence, it becomes necessary to examine data from the viewpoint of Africans as subjects and human agents rather than as objects in a European context. This implies that Afrocentricity has implications for indigenous African culture. Afrocentricity draws on research from an African viewpoint and creates Africa’s own intellectual perspective. It studies Africa as the cultural centre that is utilised in the study of African experiences, such that interpretations of data are drawn from the African perspective and in this case from a rural Zimbabwean perspective. The Afrocentric paradigm provides methods and practices that African people can use for making sense of their everyday experiences from an indigenous African point of view. Hence, a study of this nature requires an Afrocentric perspective in order to sufficiently present indigenous adaptation measures and the use of IKS.

### Objective of the study

The objective of this study is to examine indigenous adaptation measures in response to climate change that are developed and used by the community as well as measures used to sustain indigenous practices.

## Methodology

### Study area

**FIGURE 1:**
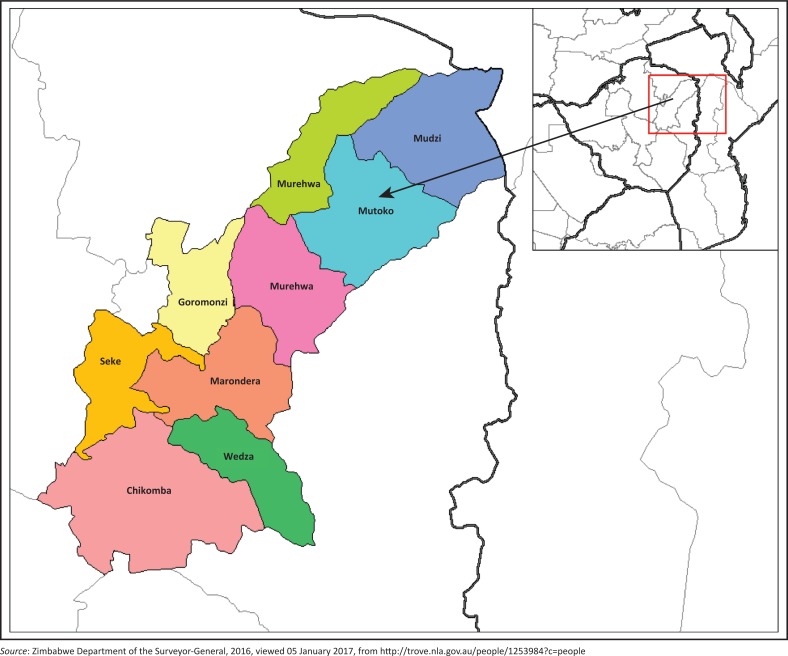
Geographical location of Mutoko rural community (towns and provinces of Zimbabwe, Mashonaland East Province, location of Mutoko and the geographical layout of Mutoko).

Mutoko is a district of Mashonaland East Province, Zimbabwe, in southern Africa (see Figure 1). It is located in the eastern part of Zimbabwe, and covers 4092.5 km^2^ (Mvumi, Donaldson & Mhunduru 1988). According to the 2012 population census, Mutoko has 146 127 inhabitants (Moyo 2016). It was established as an administrative station in 1911 and it lies 143 km from Harare, the capital city of Zimbabwe. The area is occupied by the *Buja* people. The *Buja* people are said to have settled in Mutoko from *Mhingari* in what is now Mozambique. The subsistence economy of Mutoko rural community is based on conservation farming (Fanelli & Dumba 2011). The most favourable crops in the area include maize, groundnuts, vegetables, sunflowers, sorghum, cotton, pearl millet and finger millet (Mvumi et al. 1998). The climate in the area is mild, and generally warm and temperate (Fanelli & Dumba 2011). Mutoko rural district is divided into 29 wards, each consisting of six villages, with about 1000 people per village or 80–120 families (Mvumi et al. 1998).

### Research design and sampling

The researcher employed an exploratory research design. Hair et al. (2003) define exploratory research as research conducted to gain new insights and discover new ideas. Hence, a qualitative, exploratory study was designed to probe the explanations of climate change and the indigenous adaptation measures used to cope with it in Mutoko. The use of a qualitative research design enabled the researcher to have full interaction with the study participants throughout the study. Thirty participants were drawn from two villages of Mutoko rural community to collect quality and reliable data. The study sample was composed of two groups of respondents, namely Matedza and Chibeta rural community members. The two communities are representative in the sense that they share the same experiences with all other villages in Mutoko rural community. The researcher intended to focus on participants who were either aged 50 years and above or had lived in the area for at least 10 years or more. However, these variables were difficult to establish; therefore, the researcher ultimately considered all individuals who had lived in the area for a reasonable number of years and those who proved to understand how climatic conditions had changed in the area. The reason for the age restriction was to engage people who were capable of giving a comparative explanation of climate change between the period of when they were young and the time of data collection, or the changes they had noticed in the years during which they had lived in the area. Nevertheless, the age variable together with the years of living in the area (Chibeta or Matedza) was fundamental in selecting participants to become part of the study sample. A non-probability technique known as criterion purposive sampling was employed to select the study participants. Bryman (2012) points out that in criterion purposive sampling, the sample units are selected because they have particular characteristics that will enhance the exploration and understanding of the aims and objectives of the study.

### Data collection and analysis

Data were collected using in-depth interviews. The collection of data was informed by factors such as age of the respondents, period of stay in the area and general understanding of climate change and indigenous practices used in Mutoko rural community. In order to fully comprehend the use of indigenous practices to adapt to climate change by Mutoko rural community members, a qualitative research method was employed. Interviews were conducted with 30 community members in their own households. The interviews were conducted in the same pattern in both villages. Data were analysed through a thematic content analysis (TCA). Braun and Clarke (2006) define TCA as a method used for identifying, analysing and reporting patterns in data. TCA operates by breaking down the information collected into themes. The researcher identified trends and patterns that developed from the data collected. The patterns were then coded and classified into different categories that were used to analyse the use of indigenous practices to adapt to climate hazards in Mutoko rural community.

### Findings and discussion

#### Adaptation measures and indigenous practices

There are numerous adaptation measures to climate change that significantly help the community to sustain indigenous practices. It has been reported that the community no longer grows maize in large quantities; as such, most of the respondents indicated that they have shifted to millet and sorghum in order to adapt to rainfall scarcity. With millet and sorghum, they still continue brewing traditional beer which forms the basis for all traditional functions in the Shona culture. Millet compensates for maize in preparing *sadza* (thick porridge) which is their staple food. Most respondents mentioned that they had turned from growing maize to growing millet and sorghum because it is more suitable to the current climate. They emphasised that this is in line with what their forefathers used to do because they could have been told by their ancestors to grow millet or sorghum when there was not enough rainfall. Also, the respondents indicated that millet provides them with mealie-meal for *sadza*, and sorghum provides them with ingredients for traditional beer which they use for most of their functions such as *kurova guva* (veneration of the dead), *parufu* (during funerals) and *paroora* (during traditional weddings). As a result, through the adaptation measures the community employs, cultural practices are also sustained. The shift from maize to millet and sorghum by Mutoko community members supports the assertion of Jianchu et al. (2007) that local communities have developed adaptation measures relevant to their cultural values to lessen the impacts of climate threats on their livelihoods. Local communities have developed culture-based mechanisms of adaptation to harsh weather conditions that negatively impact their livelihoods (Jianchu et al. 2007). These mechanisms are complex and were developed and are used within cultures. They imply a greater dependability on the use of indigenous practices to sustain their lives (Elia et al. 2014).

The adaptation strategies employed are in response to the climatic changes the community is witnessing. The study found that rainfall has not been consistent in the recent past. That is, the rain that the area currently receives is not of the same quantity as in previous years. The respondents indicated that crops often die before harvesting because of a lack of water and extreme heat. One of the respondents commented that:

‘The changes we are witnessing in seasons is immensely visible in the sense that long back we used to receive more rains and reap more harvest but nowadays the rains are unpredictable sometimes our crops die of heat because of lack of sufficient rains.’ (Female, 45, farmer)

Some respondents also indicated that in the 1970s and 1980s they used to receive sufficient rainfall. However, beginning in the early 1990s, the amount of rain they received began to decrease. The numerous effects of climate change are also articulated by Mugambiwa and Tirivangasi (2017) who assert that:

there are numerous potential effects climate change could have on agriculture. It affects crop growth and quality and livestock health. Farming practices could also be affected as well as animals that could be raised in particular climatic areas. (p. 1)

Hence, these changes in rainfall patterns and crop growth informs the community’s decision to shift from growing maize to growing sorghum and millet, among other indigenous practices employed by the community.

The study also found that supplementary irrigation was once practiced as an adaptation strategy, but it has since failed because the rivers from which the water is collected dry up so early. Therefore, the respondents pointed out that the only adaptation strategy they had was supplementing maize with drought-resistant crops such as millet and sorghum. Some of them argued that even their forefathers used to do this and that it was easy because they could be told what to grow by ancestors through spirit mediums. The current study has also established that community members in Mutoko have developed large storage systems that accommodate their produce and help them to sustain their livelihoods for the future. To support this, one of the respondents commented that:

‘We have developed storage systems where we keep our crops in large quantities. With these storage systems we are supposed to go for a long time with our produce. But since we are having small harvests due to climate change we have turned to growing millet and sorghum.’ (Male, 68, farmer)

Asante (1980) stipulates that in Afrocentricity, history and culture form the foundation of human activities and life in general. As a result, the use of various adaptation measures used by the community is meant to restore the way of life and cultural activities that their forefathers used to perform. In the African context, culture-based mechanisms that are implemented to adapt to climate change are passed from generation to generation. As a result, in so doing the community will be observing their history. In support of this, Adeleke (2010) argues that Afrocentricity draws its orientation from the history of the African subject. Most of the respondents indicated that the community had turned to growing millet and sorghum instead of maize because with maize they constantly get low harvests as it often dries before it is ripe. However, with millet and sorghum they get good harvests even though they receive low rainfall and have extremely hot days. The respondents acknowledged that they have considered millet and sorghum to be a perfect alternative because these crops enable the community to continue practicing their activities, for example, during ceremonies they use sorghum to prepare traditional beer, and the *sadza* that people eat will be from millet. The study has revealed that even though there are numerous adaptation strategies available to adapt to climate change, the Mutoko community largely relied on indigenous strategies. These strategies are in line with Afrocentricity which theoretically informed the study.

#### Adaptation to the impact of climate change

Respondents provided strategies that they use to adapt to climate change. These strategies include mulching, burning trees and using the charcoal as manure, tilling the land before the rains and creating temporary walls on riverbanks in order to store water for irrigation. It is explicit that most of these adaptation strategies are community-based mechanisms. These mechanisms are a response to the changing climatic conditions which, for example, result in rivers drying up early and prematurely. Some respondents pointed out that rivers are now drying up earlier than they used to in the past. One respondent commented that:

‘It is worrying that rivers are now drying up so early. This has serious consequences because we need water for domestic use, livestock and irrigation. It has become a norm in our community that from June through November we struggle for water and often queue for water at the local bore hole.’ (Female, 65, farmer)

Given the challenges presented by the respondents, the community has been made to invent measures that enable them to survive climate change. Consequently, most respondents acknowledged that numerous changes had taken place in the climate and what they were experiencing now was quite different from when they were young. The use of indigenous practices significantly helped them in adapting to the changing environment. Adeleke (2010) is of the view that Afrocentricity is determined to reclaim ancient African civilizations as the basis of interpreting and understanding ways of life of African peoples; that is, their narratives, daily activities and culture. Interestingly, all the adaptation measures employed by the community were from an African perspective. Most of the respondents indicated that since they now receive less rain, which often comes late, they use various strategies in their farming activities. These strategies include what they call *mujogo* in their local language. *Mujogo*, which is slightly related to muching, is a process whereby they dig holes for crops; when they drop the seed it is covered with dry grass and water in order to fast-track the growing process of the plant. Apart from *mujogo*, the community also burns trees around their farmlands and they use the charcoal from the burnt trees as manure for their crops. They also make good use of riverside wells to sustain their crops. The other strategy that the respondents now employ is the tilling of land before rainfall in preparation for agricultural activities. One of the respondents pointed out that:

‘We have adopted a system of tilling the land before rain comes so that by the time it comes the soil will be able to hold water for a long time. Also we are now utilizing crops like millet so that we can get good harvests.’ (Male, 70, farmer)

The respondents mentioned that apart from tilling the land before rainfall they also store water for agricultural purposes by building temporary walls around riverbanks. When the river dries up they are then able to sustain their crops through irrigation from riverbank wells. Generally, the respondents demonstrated that even though climate change had numerous impacts on their livelihoods, they equally had workable strategies. It was discovered that these strategies were unique and have been sustained for a long time. The view that Afrocentricity implies that Africans should be studied from their own worldview is largely upheld on this aspect. The uniqueness of the strategies employed by community members necessitates this study to be informed by Afrocentricity. In a study by Nkomwaa et al. (2014) on IKS and climate change adaptation strategies in agriculture in the Chagaka area, Malawi, farmers have always utilised a variety of traditional aspects in order to enhance their farming practices. Also, Gyampoh et al. (2014) observed that rural communities that are vulnerable to climate change have strong adaptive capacities. Adapting to drought, scarcity of rain and decreased production of crops is accomplished through community-based measures to sustain human livelihoods (Gyampoh et al. 2014). In the current study, most farmers reported that they changed crop types from maize to sorghum and millet in order to cope with climate change. This is in line with a study conducted in Pakistan by Abid et al. (2016) which shows that farmers opted to change crop types because of an incidence of heavy pest and insect attacks, soil problems and extreme temperature events. Some farmers reported that they had replaced cotton crops with maize crops since 2010 because of cotton’s susceptibility to heavy pest attacks and changing weather conditions (Abid et al. 2016). Even though subsistence farmers have always adopted adaptive strategies to some of these changes over the years, effective adaptation strategies should be aimed at securing communities’ well-being in the face of climatic changes (Somah 2013).

## Conclusion

In conclusion, the study has established that climate change adaptation measures employed by the community of Mutoko rural district have significantly helped them to sustain their indigenous practices in many spheres of life. Also, the use of IKS through activities such as crop type change from maize to traditional millet and sorghum, which facilitates their traditional lifestyle, re-establishes the community’s indigenous practices since they are made to return to old practices. As a result, even though climate change adaptation is not directly meant to sustain indigenous practices, it is apparent that the unintended outcome of adaptation in the community is sustaining indigenous practices. This study also discovered that the use of IKS to adapt to climate change is the common adaptation method and widely employed by the community. As a result, the method reduces the vulnerability of society to changes in the climate system. From an Afrocentricity stand point, the current study triumphed in achieving its objective. Asante (1993) argued that Afrocentricity has to do with the need for African people to be relocated historically, economically, socially, politically and philosophically. In that regard, adaptation measures to sustain indigenous practices and the use of IKS to adapt to climate change in Mutoko rural district was explicitly and implicitly explored from the community’s worldview and culture. Consequently, understanding indigenous adaptation methods employed by the community will significantly help to moderate and mitigate climate change impacts.

## References

[CIT0001] Abid M., Schilling J., Scheffran J. & Zulfiqar F., 2016, ‘Climate change vulnerability, adaptation and risk perceptions at farm level in Punjab, Pakistan’, *Science of the Total Environment* 547, 447–460. https://doi.org/10.1016/j.scitotenv.2015.11.125 26836405 10.1016/j.scitotenv.2015.11.125

[CIT0002] Adeleke T., 2010, ‘Against Euro-Cultural Hegemony: Black Americans, Afrocentricity and globalization’, in Ommundsen W., Leach M. & Vandenberg A. (eds.), *Cultural citizenship and the challenges of globalization*, pp. 225–244, Putnam Press, Cresskill, NJ.

[CIT0003] Akoh B., Bizikova L., Parry J., Creech H., Karami J., Echeverria D. et al., 2011, *Africa transformation-ready: The strategic application of information and communication technologies to climate change adaptation in Africa (Final Report for the African Development Bank, the World Bank and the African Union)*, International Institute for Sustainable Development, Bristol.

[CIT0004] Asante M.K., 1980, *The painful demise of Afrocentrism: An Afrocentric response to critics*, Africa World Press, Trenton, NJ.

[CIT0005] Asante M.K., 1993, *Classical Africa* [part of the Asante Imprint series of high school textbooks], Peoples Publishing Group, Maywood, NJ.

[CIT0006] Beck U., 1992, *Risk society: Towards a new modernity*, Sage, London.

[CIT0007] Bradshaw B., Dolan H. & Smit B., 2004, ‘Farm-level adaptation to climatic variability and change: Crop diversification in the Canadian prairies’, *Climate Change* 67, 119–141. https://doi.org/10.1007/s10584-004-0710-z

[CIT0008] Braun V. & Clarke V., 2006, ‘Using thematic analysis in psychology’, *Qualitative Research in Psychology* 3, 77–101. https://doi.org/10.1191/1478088706qp063oa

[CIT0009] Brenkert A. & Malone E., 2005, ‘Modelling vulnerability and resilience to climate change: A case study of India and Indian States’, *Climatic Change* 72, 57–102. https://doi.org/10.1007/s10584-005-5930-3

[CIT0010] Brooks N. & Adger W.N., 2005, *Assessing and enhancing adaptive capacity. Adaptation policy Frameworks for climate change: Developing strategies, policies and measures*, Cambridge University Press, Cambridge.

[CIT0011] Bhusal Y., 2009, *Local people’s perceptions on climate change, its impacts and adaptation measures in Mid-Mountain Region of Nepal*, [*A Case study from Kaski District]*, Tribhuvan University Institute of Forestry, Nepal.

[CIT0012] Bryman A., 2012, *Social science research methods*, 4th edn., Oxford University Press, Oxford.

[CIT0013] Burton I., Smith J.B. & Lenhart S., 1998, ‘Adaptation to climate change: Theory and assessment’, in Feenstra J.F., Burton I., Smith J.B. & Tol R.S.J. (eds.), *Handbook on methods for climate change impact assessment and adaptation strategies*, pp. 880–912, UNEP, Nairobi, Kenya.

[CIT0014] Burton I., 1992, *Adapt and thrive*, Canadian Climate Centre, unpublished manuscript, Toronto, ON.

[CIT0015] Denevan W.M., 1983, ‘Adaptation, variation and cultural geography’, *Professional Geographer* 35 (4), 399–406. https://doi.org/10.1111/j.0033-0124.1983.00399.x

[CIT0016] Elia E.F., Mutala S. & Stilwell C., 2014, ‘Indigenous knowledge use in sessional weather forecasting in Tanzania: The case of semi-arid central Tanzania’, *South African Journal of Library and Information Sciences* 80, 1 https://doi.org/10.7553/80-1-1395

[CIT0017] Eriksen S., O’Brien K. & Rosentrater L., 2008, *Climate change in Eastern and Southern Africa: Impacts, vulnerability and adaptation*, Global Environmental Change and Human Security (GECHS) Report 2008:2, University of Oslo, Oslo, Norway.

[CIT0018] Fanelli C.W. & Dumba L., 2011, *Conservation farming in rural Zimbabwe*, AgriCultures Network, viewed 24 May 2016, from http://www.agriculturesnetwork.org/

[CIT0019] Ford J.D., 2012, ‘Indigenous health and climate change’, *American Journal of Public Health* 102(7), 1260–1266. https://doi.org/10.2105/AJPH.2012.300752 22594718 10.2105/AJPH.2012.300752 PMC3477984

[CIT0020] Gbetibouo G.A., 2008, *Understanding farmers’ perceptions and adaptation to climate change and variability: The case of Limpopo Basin, South Africa*, Policy Brief 15–18, International Food Policy Research Institute, Pretoria.

[CIT0021] Global Humanitarian Forum (GHF), 2009, *Human impact report 2009*, viewed 10 April 2016, from http://www.ghf-ge.org/human-impact-report.pdf

[CIT0022] Gwimbi P., 2009, ‘Cotton farmers’ vulnerability to climate change in Gokwe District (Zimbabwe): Impact and influencing factors’, Jàmbá: *Journal of Disaster Risk Studies* 2(1), 81–92. https://doi.org/10.4102/jamba.v2i2.17

[CIT0023] Gyampoh B.A., Amisah S., Idinoba M. & Nkem J., 2014, ‘Using traditional knowledge to cope with climate change in rural Ghana’, *Proceedings, Third International Conference on Climate and Water*, pp. 205–213, Finnish Environment Institute (SYKE), Helsinki, Finland.

[CIT0024] Hair B., Babin B.J., Money A.H. & Samouel P., 2003, *Essentials of business research*, Wiley, New York.

[CIT0025] Hisali E., Birungi P. & Buyinza F., 2011, ‘Adaptation to climate change in Uganda: Evidence from micro level data’, *Global Environmental Change* 21, 1245–1261. https://doi.org/10.1016/j.gloenvcha.2011.07.005

[CIT0026] Jianchu X., Shrestha A., Rameshananda V.R., Eriksson M. & Hewitt K., 2007, *Regional challenges and local impacts of climate change on mountain ecosystems and livelihoods*, ICIMOD Technical Paper, Nepal International Centre for Integrated Mountain Development (ICIMOD), Kathmandu, Nepal.

[CIT0027] Kershaw T., 1990, ‘The emerging paradigms in Black studies’, in Anderson T. (ed.), *Black studies: Theory, methods, and cultural perspectives*, pp. 17–24, Washington State University Press, Pullman, WA.

[CIT0028] Keto C.T., 1995, ‘Vision, identity and time: The Afrocentric paradigm and the study of the past’, Kendall/Hunt, Dubuque, IA.

[CIT0029] Leary N., Adejuwon J., Barros V., Batimaa P. Biagini B., Burton I. et al., 2007, *A stitch in time: Lessons for climate change adaptation from the AIACC Project*, AIACC Working Paper 48, The AIACC Project Office, International START Secretariat, Washington, DC.

[CIT0030] Madzwamuse M., 2010, ‘Climate change vulnerability and adaptation preparedness in South Africa’, Heinrich Böll Stiftung South Africa, Cape Town.

[CIT0031] Mano R. & Nhemachena C., 2007, *Assessment of the economic impacts of climate change on agriculture in Zimbabwe: A Ricardian approach*, Policy Research Working Paper 4292, The World Bank, Washington, DC.

[CIT0032] Ministry of Environment and Tourism, 2006, *National capacity self-assessment*, Government of Zimbabwe, Harare, viewed 07 May 2016, from http://www.undp.org/content/undp/en/home/librarypage/environmentenergy/integrating_environmentintodevelopment/ncsa-final-reports--action-plans-andccr.html

[CIT0033] Mkabela Q., 2005, ‘Using the Afrocentric method in researching indigenous African culture’, *The Qualitative Report* 10(1), 178–189.

[CIT0034] Moyo A., 2016, ‘Dry times in Mutoko district’, *The Sunday Mail*, 06 March 2016, viewed 24 May 2016, from http://www.sundaymail.co.zw/

[CIT0035] Mugambiwa S.S. & Tirivangasi H.M., 2017, ‘Climate change: A threat towards achieving “Sustainable Development Goal number two” (end hunger, achieve food security and improved nutrition and promote sustainable agriculture) in South Africa’, *Jàmbá: Journal of Disaster Risk Studies* 9(1), 1–7. https://doi.org/10.4102/jamba.v9i1.350 10.4102/jamba.v9i1.350 PMC6014178 29955332

[CIT0036] Mvumi B., Donaldson T. & Mhunduru J., 1988, *A report on baseline data available for Mutoko District, Mashonaland East Province*, University of Zimbabwe, Harare.

[CIT0037] Nhemachena C. & Hassan R., 2007, *Micro-level analysis of farmers’ adaptation to climate change in Southern Africa*, IFPRI Discussion Paper No. 00714, IFPRI, Washington, DC.

[CIT0038] Nkomwaa E.C., Joshua M.K., Ngongondo C., Monjerezi M. & Chipungu F., 2014, ‘Assessing indigenous knowledge systems and climate change adaptation strategies in agriculture: A case study of Chagaka Village, Chikhwawa, Southern Malawi’, *Physics and Chemistry of the Earth* 69, 164–172. https://doi.org/10.1016/j.pce.2013.10.002

[CIT0039] Rankoana S.A., 2016, ‘Perceptions of climate change and the potential for adaptation in a rural community in Limpopo Province, South Africa’, *Sustainability* 8, 672 https://doi.org/10.3390/su8080672

[CIT0040] Smit B., Burton I., Klein R.J.T. & Wandel J., 2000, ‘The anatomy of adaptation to climate change and variability’, *Climate Change* 45, 223–251. https://doi.org/10.1023/A:1005661622966

[CIT0041] Smit B. & Wandel J., 2006, ‘Adaptation, adaptive capacity and vulnerability’, *Global Environmental Change* 16, 282–292. https://doi.org/10.1016/j.gloenvcha.2006.03.008

[CIT0042] Smith J.B., Ragland S.E. & Pitts G.J., 1996, ‘A process for evaluating anticipatory adaptation measures for climate change’, *Water, Air and Soil Pollution* 92, 229–238. https://doi.org/10.1007/978-94-017-1053-4_21

[CIT0043] Somah T.P., 2013, ‘Climatic change impacts on subsistence agriculture in the Sudano-Sahel zone of Cameroon – Constraints and opportunities for adaptation’, PhD thesis, Brandenburgische Technical University.

[CIT0044] Szerszynski B., 2010, ‘Reading and writing the weather: Climate technics and the moment of responsibility’, *Theory, Culture & Society* 28, 2–3. https://doi.org/10.1177/0263276409361915

[CIT0045] Tambo J.A., 2016, ‘Adaptation and resilience to climate change and variability in north-east Ghana’, *International Journal of Disaster Risk Reduction* 17, 85–94. https://doi.org/10.1016/j.ijdrr.2016.04.005

[CIT0046] UNFCCC, 2007, *Climate change: Impacts, vulnerabilities and adaptation in developing countries*, United Nations Framework Convention on Climate Change, Bonn, Germany.

[CIT0047] Urry J., 2010, ‘Consuming the planet to excess’, *Theory, Culture & Society* 27, 2–3. https://doi.org/10.1177/0263276409355999

[CIT0048] Wahaa K., Muller C., Bondeau A., Dietrich J.P., Kurukulasuriya P., Heinke J. et al., 2013, ‘Adaptation to climate change through the choice of cropping system and sowing date in sub-Saharan Africa’, *Global Environmental Change* 23, 130–143. https://doi.org/10.1016/j.gloenvcha.2012.11.001

[CIT0049] Yohe G. & Tol R., 2002, ‘Indicators for social and economic coping capacity: Moving toward a working definition of adaptive capacity’, *Global Environmental Change* 12, 25–40. https://doi.org/10.1016/S0959-3780(01)00026-7

[CIT0050] Ziervogel G., Cartwright A., Tas A., Adejuwon J., Zermoglio F., Shale M. et al., 2008, *Climate change and adaptation in African agriculture*, Stockholm Environment Institute, New York.

[CIT0051] Zimbabwe Department of the Surveyor-General, 2016, viewed 05 January 2017, from http://trove.nla.gov.au/people/1253984?c=people

